# Relationship between pulmonary artery hemodynamics and right ventricle function in pulmonary arterial hypertension using cardiovascular magnetic resonance

**DOI:** 10.1186/1532-429X-14-S1-P82

**Published:** 2012-02-01

**Authors:** El-Sayed Ibrahim, Abubakr Bajwa

**Affiliations:** 1 Department of Radiology, University of Florida, Jacksonville, FL, USA; 2 Department of Medicine, University of Florida, Jacksonville, FL, USA

## Summary

A comprehensive CMR exam was developed for evaluating right ventricle (RV) and pulmonary artery (PA), and was tested on 25 PA hypertension (PAH) patients. Standard non-CMR measurements were also collected. Comprehensive statistical analysis was conducted to study relationships between parameters, parameters’ significance, and data redundancy and reduction. The results showed strong correlations between most RV and PA parameters; and between most MRI parameters and gold standard PA pressure. In conclusion, RV and PA are coupled and affected in PAH. Both entities should be evaluated and interpreted together.

## Background

Pulmonary artery (PA) hypertension (PAH) is characterized by elevated PA pressure (PAP), which increases right ventricular (RV) afterload. Although PAP is the gold standard for evaluating PAH, it is the RV condition that predicts patient survival. However, there is insufficient data about the relationship between RV and PA in PAH. The goal of this study is to investigate this relationship using CMR.

## Methods

25 PAH patients, confirmed by cath, were scanned on 3T Siemens scanner. A comprehensive CMR exam was developed (~30 minutes) that included cine, strain-encoded (SENC), and flow (across main PA (MPA) and tricuspid valve) images. Image processing was conducted to obtain these parameters: Cine (RV cardiac index (CI), LV EF, ventricular vol index (VVI), ventricular mass index (VMI), right atrium (RA) size, MPA diameter, lunar index (LI) (Fig.1)); SENC (RV longitudinal strain and strain rate); Tricuspid Flow (RV early-to-atrial (E/A) filling); and PA Flow (pulse wave velocity (PWV), distensibility, accel-to-eject (a/e) time, flow rate, mean velocity). Mean PAP (mPAP), pulmonary vascular resistance (PVR), tricuspid jet velocity (Tri JV), 6 minute walk (6MW), and brain natriuretic peptide (BNP) results were obtained. Parameters were divided into 3 groups: RV (CI, VVI, VMI, LI, RV E/A, strain, strain rate, LVEF, RA size); PA (MPA diameter, PWV, distensibility, a/e time, flow rate, mean velocity, PA diameter); and Measurements (mPAP, PVR, Tri JV, 6MW, BNP). Comprehensive statistical analysis included: Correlation (within- and across-groups); Regression (identify significant parameters, investigate redundancy, study relationship between CMR parameters and PAP); and Principal Component Analysis (PCA) (data reduction). Multiple regression used backward deletion (mPAP is dependent variable).

## Results

Fig.2 shows color-coded correlation map between all parameters, which shows different degrees of correlations. Correlation analysis results came in agreement with regression and PCA analyses. Strongly correlated within-group parameters reflected data redundancy. Weakly correlated parameters reflected parameters’ non-specificity. PA parameters showed strong correlations with (VVI, VMI, LI, strain, strain rate); moderate correlations with (CI); and weak correlations with (RV E/A, LVEF, RA size). In regression analysis, the following parameters were eliminated in this order: RV group (RV E/A, RA size, LVEF, CI, strain rate, VVI); PA group (distensibility, a/e time, MPA diameter); both PA and RV groups for estimating mPAP (LVEF, velocity mean, distensibility, RA size, a/e time, RV E/A, strain rate, VMI, LI, PWV). PCA analysis resulted in: RV group (2 components, variances=56.9%,14.9%); PA group (1 component, variance=76.8%); Measurements (2 components, variances=61.7%,20.2%).

## Conclusions

RV and PA are coupled and their functions are affected in PAH. Both entities should be evaluated and interpreted together for more understanding of disease pathophysiology and better diagnosis and treatment.

## Funding

James & Esther King Grant # 09KN-03-23138.

